# Factors associated with pre-ART loss-to-follow up in adults in rural KwaZulu-Natal, South Africa: a prospective cohort study

**DOI:** 10.1186/s12889-016-3025-x

**Published:** 2016-04-27

**Authors:** Michael Evangeli, Marie-Louise Newell, Nuala McGrath

**Affiliations:** Department of Psychology, Royal Holloway University of London, Egham Hill, Egham, Surrey, London, TW20 0EX UK; Africa Centre for Health and Population Studies, University of KwaZulu-Natal, Somkhele, South Africa; Academic Unit of Health and Development, Faculty of Medicine, University of Southampton, Southampton, UK; Academic Unit of Primary Care and Population Sciences and Department of Social Statistics and Demography, University of Southampton, Southampton, UK

**Keywords:** HIV, Pre-ART, Loss-to-follow up, South Africa, Psychosocial

## Abstract

**Background:**

Timely initiation of antiretroviral treatment (ART) requires sustained engagement in HIV care before treatment eligibility. We assessed loss to follow-up (LTFU) correlates in HIV-positive adults accessing HIV treatment and care, not yet ART-eligible (CD4 >500 cells/mm^3^).

**Methods:**

This was a sub-study of a prospective cohort study (focusing on sexual behaviour) in an area of high HIV prevalence and widespread ART availability in rural KwaZulu-Natal, South Africa. Psychosocial, clinical and demographic data were collected at recruitment from individuals with CD4 >500 cells/mm^3^. LTFU was defined as not attending clinic within 13 months of last visit or before death. Individuals starting ART were censored at ART initiation. Data were collected between January 2009 and January 2013. Analysis used Competing Risks regression.

**Results:**

Two hundred forty-seven individuals (212 females) were recruited (median follow-up 2.13 years, total follow-up 520.15 person-years). 86 remained in pre-ART care (34.8 %), 94 were LTFU (38.1 %), 58 initiated ART (23.5 %), 7 died (2.8 %), 2 transferred out (0.8 %). The LTFU rate was 18.07 per 100 person-years (95 % CI 14.76–21.12). LTFU before a competing event was 13.5 % at one and 34.4 % at three years. Lower LTFU rates were significantly associated with age (>37 versus ≤37 years: adjusted sub-Hazard ratio (aSHR) 0.51, 95 % CI 0.30–0.87), openness with family/friends (a little versus not at all, aSHR 0.81, 95 % CI 0.45–1.43; a lot versus not at all, aSHR 1.57, 95 % CI 0.94–2.62), children (0 versus 4+, aSHR 0.68, 95 % CI 0.24–1.87; 1 versus 4+, aSHR 2.05 95 % CI 1.14–3.69, 2 versus 4+; aSHR 1.71, 95 % CI 0.94–3.09; 3 versus 4a, aSHR 1.14, 95 % CI 0.57–2.30), previous CD4 counts (1 versus 0, aSHR 0.81, 95 % CI 0.45–1.43; 2+ versus 0, aSHR 0.43, 95 % CI 0.25–0.73), and most recent partner HIV status (not known versus HIV-positive, aSHR 0.77, 95 % CI 0.50–1.19; HIV-negative versus HIV-positive, aSHR 2.40, 95 % CI 1.18–4.88). The interaction between openness with family/friends and HIV partner disclosure was close to significance (*p* = 0.06). Those who had neither disclosed to partners nor were open with family/friends had lowest LTFU rates.

**Conclusions:**

Strategies to retain younger people in pre-ART care are required. How openness with others, partner HIV status and disclosure, and children relate to LTFU needs further exploration.

## Background

With an estimated 6.4 million people, South Africa has the world’s largest HIV-positive population [1]; among adults aged 15–49, HIV prevalence is estimated at 18.6 %. South Africa’s public sector antiretroviral (ART) programme began in 2004 and by the end of 2012 over two million were on treatment [1]. Even with expanded global ART guidelines [2], however, significant numbers remain ART-ineligible in countries such as South Africa not currently recommending treatment for all people living with HIV. Retention in care in this group is important for timely future ART initiation. Understanding loss-to-follow-up (LTFU) (non-attendance at scheduled clinic visits) from pre-ART care is also important for test-and-treat interventions [3].

Despite lower mortality rates, overall attrition (combined LTFU, death and transfer to another programme) for those ART ineligible is higher than for those ART-eligible [4]. Median attrition rates of 54 % (across studies with follow-ups from 7 months to 5 years) have been reported in sub-Saharan Africa (SSA) [5, 6]. Most studies have used a cut-off of CD4 <200 cells/mm^3^ for ART-eligibility [4] although South Africa guidelines for ART initiation in South Africa rose to CD4 counts of <500 cells/mm^3^ in January 2015, consistent with WHO guidelines at that time [7].

There have been few studies of LTFU correlates in SSA for individuals not yet ART-eligible, particularly those with a CD4 count of >500 cells/mm^3^. A clearer understanding of LTFU in this group may help develop targeted interventions to enhance programme retention. In South Africa, one study examined LTFU in adults (*n* = 4223, 84 % female) 13 months after an initial CD4 count of ≥200 cells/mm^3^ [8]. Higher LTFU levels were independently related to being employed, male, younger age, higher initial CD4 count, out-migration and greater household size. Thirty five per cent of individuals with an initial CD4 count of >500 cells/mm^3^ were retained in care. Elsewhere in South Africa [9], among 356 individuals newly enrolled in pre-ART care (CD4 count ≥250 cells/mm^3^), higher LTFU levels at one year were related to unemployment and higher initial CD4 count. As LTFU was more common in those with higher CD4 counts in both of these studies, there is a need to assess correlates of engagement in individuals before symptoms develop. In a third study, in Kenya [10], with 530 individuals with an initial CD4 count of ≥200 cells/mm^3^, higher LTFU levels at six months after registration in HIV care, were related to greater distance from a main road and being unmarried.

We were unable to find research on *psychosocial* correlates of LTFU in pre-ART care. Understanding these relationships may enable researchers to suggest *why* distal factors such as gender and employment are associated with LTFU. Psychosocial variables may be more amenable to intervention than structural or demographic factors. We recently found that, in those ART-eligible, higher LTFU rates were related to male sex, social support (*increased* openness and *less* reliance on family and friends), social capital (believing that community problems would be solved at higher levels, e.g., traditional and district leaders rather than individuals and neighbours), younger age and having children [11]. Predictors of LTFU in those not yet eligible for ART may be different.

We used data from a prospective cohort study of individuals recruited from HIV care clinics with a CD4 count of ≥500 cells/mm^3^ and not yet ART-eligible, in an area of high HIV prevalence and widespread ART availability in KwaZulu-Natal, South Africa [12, 13] to explore the associations between psychosocial, demographic and clinical variables and LTFU.

## Methods

### Study design and location

The study used a prospective cohort design [12] with recruitment between January 2009 and April 2011 and follow-up until January 2013. It took place in the Hlabisa sub-district of uMkhanyakude, in rural northern KwaZulu-Natal, South Africa, an area with an HIV adult prevalence estimate of 24 % [14]. One third of this sub-district covers the Africa Centre Demographic Surveillance Area (DSA) (http://www.africacentre.ac.za/).

The HIV treatment and care programme began in 2004 and is large scale and decentralized [15]. It implements national HIV treatment guidelines, which until April 2010 denoted ART-eligibility at CD4 count ≤200 cells/mm^3^ or WHO stage 3 or 4 [16], between April 2010 and August 2011, CD4 count ≤350 cells/mm^3^ for pregnant women, active TB, WHO stage 3 or 4 condition [17], and from August 2011 until the end of the study period in January 2013, CD4 count <350 cells/mm^3^, MDR-TB patients, and all HIV positive pregnant or breastfeeding women [18]. Within the sub-district, sharing household membership or living arrangements with individuals in HIV treatment and care is common [19], with HIV disclosure to an average of four family and friends for women and just over three family and friends for men [20].

Pre-ART care at the time of the study included CD4 count testing, individual counselling (with advice on healthy living, disclosure, partner notification and testing, transmission risk reduction and family planning) and peer support groups [8]. National guidelines at the time of the study recommended that individuals with CD4 counts of ≥500 cells/mm^3^ should attend clinic every 12 months for repeat clinical assessment and CD4 counts [21]. Practice varied, however, and often the study clinics advised return after 6 months [8].

### Participants

Participants were HIV-positive individuals taking part in a prospective cohort study [12] and (a) with CD4 count ≥500 cells/mm^3^ at the time of recruitment and thus not yet eligible for treatment [16–18] (b) ≥18 years (c) attending one of three study HIV clinics.

Potential participants were excluded if they were currently pregnant (as the primary focus of the prospective cohort study [12] was on sexual behaviour), planned to leave the area within 12 months or had previous ART use for ≥2 weeks. All individuals meeting the inclusion criteria were approached. Individuals were recruited after receiving a CD4 count of ≥500 cells/mm^3^. Written consent was given for participation with separate consent to link study data with HIV treatment and care programme and surveillance data.

### Measurement of variables

Analysis used variables from (1) a baseline interview at recruitment: questions were translated into isiZulu and backtranslated into English to ensure equivalence of meaning [12, 13], (2) routinely held programme data held in a monthly updated database and (3) surveillance data: demographic information collected biannually from households and individuals and entered into a database for the approximately 90, 000 individuals within the area [22].

Variables were chosen due to their potential association with LTFU. The main psychosocial variables from the baseline interview were:

1. *Stigma*
  Twenty-four questions adapted from Sayles et al. [23] (e.g., *‘I feel ashamed to tell people that I have HIV’*). Scores were added to form a scale with a total score (out of 72) (α = 0.75).
2. *Social support*
  Five questions, derived from Myer et al. [24], covering frequency of contact with, and reliance on, family members/friends, personal disclosure to friends/family, and the availability of confidants. They were considered as separate questions due to differences in response options and low inter-item correlations.
3. *Social capital*
  This refers to an individual’s connections (structural) and trust/reciprocity (cognitive) with others [25]. Questions were based on Pronyk et al. [26]. Three *structural* questions asked about frequency of time spent with neighbours, frequency of neighbourhood crime and community group participation. Two *cognitive* questions asked about neighbours’ commitment to community projects, and problem-solving for community problems. These questions were considered separately due to differences in response options and low inter-item correlations.
4. *Antiretroviral therapy*
  - Personal knowledge of others taking ART (one question)
  - HIV optimism. One question (adapted from Elford et al. [27]) - ‘*I am less worried about HIV now that treatment is available*’.
  - ART/HIV knowledge. Eight questions (e.g., ‘*Sometimes ART can cause side effects that make people feel worse’*). Scores were added to form a scale with a total score (out of 24) (α = 0.55).
5. *Relationship quality*
  Ten questions, adapted from the 24-item Social Provision Scale [28], were used for those in a current relationship (α = 0.66).

#### Other variables collected on recruitment

The following were recorded from the baseline interview: age, gender, time since HIV diagnosis, marital status, religious affiliation and importance, most recent partner characteristics (age differential, place of residence, HIV disclosure to partner and HIV status), government grants, number of current sexual relationships, employment, clinic, extent of HIV disclosure (number of categories of people disclosed to, e.g. partner, friend, family), HIV partner disclosure, and reason for HIV testing (responses grouped into (a) self-initiated: non-sickness, e.g., *wanted to know status* (b) self-initiated sickness, e.g., *having symptoms* (c) other-initiated, e.g., *tested at antenatal clinic*). Highest educational level, migration and number of children were recorded from the surveillance database and CD4 count at recruitment (converted into quartiles) and number of previous CD4 counts were recorded from the programme database.

### Outcomes

The programme database was used to define outcomes, with verification by cross-checking with surveillance and study databases. Participants were LTFU if they had not attended an HIV treatment and care programme clinic (a) in the last 13 months (i.e., more than one month late for scheduled CD4 re-testing), or (b) in the 13 months before death, and had not transferred out of the programme. Attendance could be at any of the 17 primary health care facilities within the programme. The entry date was the date of recruitment. The end of observation date was the ART initation date, the death date (if LTFU criteria were not met or ART had not been started before death) or the last clinic date for those transferred out. Those retained in the programme were censored at the study end date (13th January 2013).

Two methods were considered to calculate the end of observation date for those LTFU (both used in previous studies [10, 29, 30]):

1. Censoring at last clinic date (last clinic date method)
2. Censoring at the midpoint between last clinic date and next scheduled clinic date, i.e, estimating a mean *actual* LTFU date (midpoint method).

Thirty-four participants last attended clinic before their baseline interview date. That is, these individuals did not return to clinic for further CD4 cell count testing but did return for their baseline interview shortly after they had received their CD4 result that determined their treatment ineligibility (median duration between last clinic date and baseline interview for this group: 14 days, interquartile range (IQR) 13–29.5). These participants would have been ineligible for analysis using the last clinic date method because their baseline interview was after their last clinic visit for CD4 testing, with a resulting loss of statistical power. As a consequence we used the midpoint method as this LTFU definition allowed their end of observation date to be after the baseline interview date.

### Analysis

Analysis used STATA 11 [31]. Distributions of four quantitative variables were examined for normality, using skew tests. ART knowledge and relationship quality were skewed (*p* < 0.01) and thus categorised. Age was also skewed (*p* < 0.01) and considered both as age bands and as a binary variable (≤37 and >37) in univariable analysis (as the upper two age bands and the lower age bands had similar estimates). Stigma was normally distributed (*p* = 0.15) but this variable was grouped into quartiles and represented by indicators in models (as a linearity assumption did not appear reasonable after univariable analysis). We compared demographic and clinical characteristics of the 34 participants who last attended clinic before their baseline interview and the remaining 213 participants. There were no statistically significant differences in demographic or clinical variables between these groups.

Univariable associations, using Competing Risks regression, were calculated between LTFU and variables at recruitment. Competing Risks regression is an alternative to Cox regression for survival data in the presence of competing risks. This approach models the subhazard of failure events in the absence of the occurrence of competing events. Multivariable analysis was conducted, using Competing Risks Regression, with the inclusion of sex and age (≤37 and >37 years). Additional variables were added in descending order of univariable relationship with LTFU if *p* ≤ 0.15, and were retained if they improved model fit using AIC values. Competing Risks regression modelling was also carried out using backward selection and with Wald tests as the criterion for model selection. Variables included in the final model using these approaches were also included in a Cox regression multivariable model to facilitate comparison between the two methods of regression. The proportionality of subhazards was examined in the Competing Risks regression model by examining time interactions. Cumulative incidence curves were used to plot the cumulative incidence function from the Competing Risks regression model. The proportional hazards assumption was tested in the Cox regression model by examining Schoenfeld residuals [32].

## Results

There were 247 participants (212 (85.8 %) female, 35 (14.2 %) male). The median follow-up time was 2.13 years (IQR 1.12–3.00), total study follow-up time 520.15 person-years. Median age was 34 years (IQR 27–43), and median CD4 count 631.5 cells/mm^3^ (IQR 559–768 cells/mm^3^) at recruitment. Ninety-five participants (38.5 %) had not received a previous CD4 test result prior to the baseline CD4 test (median number of previous CD4 tests 1, IQR 0–2).

### Programme LTFU

**Fig. 1:**
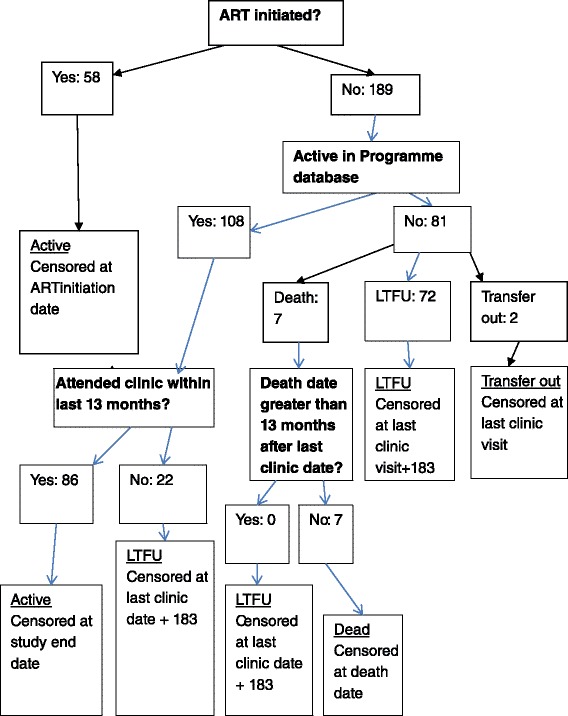
Outcome flow diagram

The process of determining LTFU outcome is presented in Fig. 1. The midpoint between the last clinic visit attended and the next scheduled clinic visit (12 months later) was 183 days*.* Ninety-four individuals were LTFU (38.1 %). The overall LTFU rate was 18.07 per 100 person-years (95 % CI: 14.76–21.12). Eighty-six individuals remained ART-ineligible but not LTFU (34.8 %), 58 initiated ART (23.5 %: median time to initiation from recruitment in this group 1.89 years: IQR 1.39–2.65), seven died (2.8 %) and two transferred out (0.8 %). LTFU before a competing event was 13.5 % at one year and 34.4 % at three years.

### Univariable analysis

Univariable analyses of associations from the Competing Risks Regression analysis between LTFU and (a) demographic and clinical variables are presented in Table 1 and (b) psychosocial variables in Table 2*.* Higher LTFU rates were related (*p* < 0.15) to number of children, age (<37 years), no group participation, fewer previous CD4 counts, increased openness to family and friends, not living with one’s most recent partner while in a relationship and the most recent partner being HIV-negative (Tables 1 and 2). Among participants living with their most recent partner, LTFU rates were similar irrespective of whether they had disclosed to them (partner disclosure: 14.23 per 100 person-years, 95 % CI 5.94–26.13; no partner disclosure: 12.46 per 100 person-years, 95 % CI 9.83–20.61).

**Table 1:** Demographic and clinical variables and loss-to-follow up in pre-ART care

		Number (% LTFU)^a^	LTFU rate (per 100 p-y)	Sub Hazard Ratio^b^(95 % CI)	*P* value of relationship with LTFU
Age in years	<27	55 (49.1)	24.24	1.00	0.07
	27–31	51(41.2)	19.55	0.74 (0.42–1.31)	
	32–37	52 (44.2)	20.98	0.83 (0.48–1.43)	
	38–44	35 (25.7)	11.20	0.41(0.19–0.89)	
	>44	54 (25.9)	12.57	0.46 (0.23–0.89)	
Age in years (binary)	≤37	158 (44.9)	21.62	1.00	0.01
	>37	89 (25.8)	12.00	0.51 (0.32–0.83)	
Age in years (continuous)				0.98 (0.96–1.00)	0.07
Sex	Female	212 (37.3)	17.29	1.00	0.40
	Male	35 (42.9)	23.72	1.27 (0.72–2.23)	
Clinic	1	109 (37.6)	17.89	1.00	0.10
	2	50 (48.0)	25.87	1.50(0.91–2.47)	
	3	88 (33.0)	14.63	0.84 (0.53–1.36)	
Marital status	Never married	199 (40.2)	19.13	1.00	0.20
	Ever Married	48 (29.2)	13.72	0.69 (0.39–1.22)	
Number of current relationships^c^	0	61 (32.8)	15.95	1.00	0.38
	≥1	185 (39.5)	18.51	1.25 (0.76–2.06)	
Religion	None	19 (31.6)	16.58	1.00	0.42
	Zionist	74 (45.9)	20.92	1.34 (0.55–3.27)	
	Shembe	48 (41.7)	22.77	1.39 (0.54–3.61)	
	Christian	90 (33.3)	15.64	0.99 (0.40–2.44)	
	Other	16 (25.0)	9.57	0.62 (0.17–2.25)	
Religion importance	Not at all	31 (32.3)	14.56	1.00	0.64
	Somewhat	49 (36.7)	16.90	1.11 (0.52–2.41)	
	Very	167 (39.5)	19.13	1.32 (0.68–2.57)	
Employment	No	198 (37.9)	18.17	1.00	0.95
	Yes	49 (38.8)	17.69	1.02 (0.61–1.70)	
Government grant (self)	No	67 (40.3)	20.26	1.00	0.59
	Yes	180 (37.2)	17.32	0.88 (0.57–1.38)	
Government grant (household)	No	94 (34.0)	15.66	1.00	0.27
	Yes	153 (40.5)	19.63	1.27 (0.83–1.94)	
HIV diagnosis	This month	31 (48.4)	24.32	1.00	0.23
	< one year	80 (41.2)	20.18	0.81 (0.44–1.48)	
	1–2 years	61 (29.5)	12.91	0.52 (0.27–1.00)	
	3 + years	75 (37.3)	18.01	0.78 (0.42–1.46)	
Highest educational level^d^	<1 year	18 (27.8)	12.28	1.00	0.07
	Primary school	67 (29.9)	13.24	1.03 (0.39–2.72)	
	Secondary, not matric	85 (35.3)	16.82	1.32 (0.52–3.36)	
	Matric and higher	67 (47.8)	23.95	2.02 (0.80–5.14)	
Number of children	None	16 (31.3)	15.71	1.25 (0.46–3.38)	0.03
	1 child	43 (53.5)	26.28	2.34 (1.32–4.14)	
	2 children	67 (44.8)	22.15	1.99 (1.13–3.50)	
	3 children	48 (33.3)	16.60	1.34 (0.69–2.60)	
	4+ children	73 (27.4)	11.84	1.00	
CD4 count	<560	62 (37.1)	19.38	1.00	0.69
	560–631	61 (42.6)	21.88	1.19 (0.69–2.07)	
	632–768	62 (32.3)	14.13	0.86 (0.48–1.56)	
	≥769	61 (41.0)	17.74	1.15 (0.65–2.01)	
Migration	No migration in last 2 years	150 (34.7)	15.84	1.00	0.32
	Migration in last 2 years	49 (42.9)	21.77	1.38 (0.82–2.28)	
	Missing data	48 (43.8)	22.01	1.36 (0.82–2.26)	
Number of previous CD4 counts	0	95 (45.3)	22.43	1.00	0.03
	1	79 (41.8)	20.11	0.89 (0.57–1.40)	
	2+	73 (24.7)	10.95	0.49 (0.29–0.85)	

^a^ *n* = 247 unless stated; ^b^Competing Risks Regression;^c^ *n* = 246; ^d^ *n* = 237

**Table 2:** Psychosocial variables and loss-to-follow up in pre-ART care

		Number (% LTFU)^a^	LTFU rate (per 100 p-y)	Sub Hazard Ratio^b^(95%CI)	*P* value of relationship with LTFU
Stigma (α = 0.75)	0–35	57 (35.1)	17.86	1.00	0.59
	36–41	54 (33.3)	14.62	0.82 (0.43–1.55)	
	42–47	64 (37.5)	16.58	0.92 (0.51–1.66)	
	48+	72 (44.4)	22.80	1.19 (0.67–2.10)	
Stigma (continuous)				1.00 (0.98–1.03)	0.83
Categories of HIV disclosure	0	34 (47.1)	25.12	1.00	0.52
	1	84 (35.7)	17.28	0.73 (0.39–1.34)	
	2	58 (41.4)	20.43	0.83 (0.44–1.57)	
	3 +	71 (33.8)	14.51	0.63 (0.34–1.19)	
HIV partner disclosure	No	91 (35.2)	17.86	1.00	0.58
	Yes	156 (39.7)	18.18	1.13 (0.73–1.74)	
Testing reason	Self-initiated: non sickness	79 (34.2)	16.96	1.00	0.82
	Self-initiated: sickness	91 (41.8)	19.83	1.17 (0.71–1.92)	
	Other initiated	77 (37.7)	17.13	1.05 (0.62–1.78)	
Changed sexual behaviour	No	78 (42.3)	20.57	1.00	0.42
	Yes	169 (36.1)	16.96	0.84 (0.55–1.28)	
Knowledge of people on ART	No	44 (45.5)	20.92	1.00	0.41
	Yes	203 (36.5)	17.43	0.82 (0.51–1.32)	
ART knowledge	Low (> = 21)	57 (40.4)	20.24	1.00	0.44
	Mid (22–23)	52 (42.3)	23.12	1.06 (0.59–1.92)	
	High (24)	138 (35.5)	15.74	0.80 (0.48–1.31)	
HIV optimism	No	79 (40.5)	20.85	1.00	0.46
	Yes	168 (31.3)	16.91	0.85 (0.55–1.31)	
Social support – time with family	< once a month/not at all	20 (35.0)	16.50	1.00	0.61
	Once a month	58 (41.4)	18.04	1.12 (0.49–2.57)	
	At least once a fortnight/several days a week	25 (28.0)	12.20	0.72 (0.26–2.03)	
	Every day	144 (38.9)	19.49	1.21 (0.55–2.65)	
Social support – time with friends	< once a month/not at all	74 (32.4)	14.38	1.00	0.49
	At least once a fortnight/Once a month	49 (46.9)	20.62	1.53 (0.87–2.69)	
	Several days a week	68 (38.2)	19.36	1.32 (0.76–2.30)	
	Every day	56 (37.5)	19.55	1.40 (0.78–2.51)	
Social support – rely on family/friends	A little/not at all	33 (36.4)	17.48	1.00	0.84
	A lot	214 (38.3)	18.16	1.06 (0.59–1.93)	
Social support – open with friends/family	Not at all	57 (36.8)	15.81	1.00	0.06
	A little	93 (31.2)	13.86	0.90 (0.52–1.57)	
	A lot	97 (45.4)	24.72	1.54 (0.93–2.55)	
Social support- confidant^c^	No	16 (43.8)	20.91	1.00	0.49
	Yes	226 (38.1)	18.05	0.74 (0.32–1.72)	
Social capital – time with neighbours	< once a month/not at all	65 (43.1)	18.80	1.00	0.88
	At least once a fortnight/Once a month	38 (34.2)	15.17	0.78 (0.41–1.49)	
	Several days a week	61 (37.7)	18.30	0.93 (0.54–1.59)	
	Every day	83 (36.1)	18.78	1.00 (0.60–1.67)	
Social capital – neighbourhood crime^d^	Common	108 (39.8)	18.85	1.00	0.38
	Unusual	87 (33.3)	14.95	0.81 (0.51–1.29)	
	Rare	49 (42.9)	22.77	1.21(0.72–2.05)	
Social capital - group participation	No	197 (41.1)	20.32	1.00	0.07
	Yes	50 (26.0)	10.69	0.58 (0.32–1.05)	
Social capital – neighbours giving time	No	63 (38.1)	16.42	1.00	0.50
	Yes	184 (38.0)	18.72	1.16 (0.75–1.82)	
Social capital – neighbours giving money	No	74 (37.8)	15.81	1.00	0.42
	Yes	173 (38.2)	19.24	1.19 (0.78–1.82)	
Social capital –community working together	Individual/neighbours or traditional leaders or municipal/district leaders taking lead	166 (34.9)	16.57	1.00	0.18
	Traditional and local leaders together taking lead	81 (44.4)	21.15	1.32 (0.88–2.00)	
Relationship quality^e^	Low (0–23)	59 (42.4)	19.01	1.00	0.91
	Mid (24–26)	68 (39.7)	19.26	0.95 (0.55–1.65)	
	High (27–30)	70 (38.6)	18.29	0.89 (0.52–1.52)	
Most recent partner age differential^f^	Older	186 (36.6)	16.80	1.00	0.40
	Same	26 (38.5)	19.87	1.08 (0.55–2.15)	
	Younger	34 (47.1)	26.21	1.45 (0.85–2.49)	
Most recent partner location	In neighbourhood	23 (43.5)	21.42	1.00	0.10
	Out of neighbourhood	109 (45.0)	22.22	1.06 (0.54–2.08)	
	With participant	115 (30.4)	13.84	0.67 (0.33–1.34)	
Most recent partner HIV disclosure	No	92 (35.9)	18.15	1.00	0.69
	Yes	155 (39.4)	18.03	1.09 (0.71–1.67)	
Most recent partner HIV status	Positive	95 (41.1)	18.95	1.00	0.10
	Not known	138 (34.1)	16.14	0.78 (0.51–1.19)	
	Negative	14 (57.1)	34.60	1.78 (0.81–3.90)	

^a^ *n* = 247 unless stated; ^b^Competing Risks Regression; ^c^ *n* = 242; ^d^ *n* = 244; ^e^ *n* = 197 (for those with a current main partner at enrolment); ^f^ *n* = 246

### Multivariable analysis

In the final main effect model from the Competing Risks regression analysis (Table 3), higher LTFU rates were independently associated with younger age, increased openness with family and friends, fewer previous CD4 counts, most recent partner status being HIV-negative and number of children. The proportionality of subhazards assumption was met. Sex was not significantly associated with LTFU in the final model (or in univariable analysis). The same final model was produced regardless of the method of variable inclusion (forward or backward) and the method of model selection (AIC values or *p* values from Wald tests). The Cox regression model using the same exposures that were retained in the Competing Risks regression model showed very similar estimates and p values. Proportional hazards assumptions were not violated for any of the variables in the final Cox regression model or for the model as a whole (*p* = 0.60).

**Table 3:** Final multivariable model of associations with loss-to-follow up in pre-ART care using Competing Risks Regression (*n* = 247)

		Subhazard Ratio (95 % CI)	P value of association with LTFU	Adjusted Subhazard Ratio (95 % CI)	*P* value of association with LTFU
Age	≤37	1.00	0.01	1.00	0.01
	>37	0.51 (0.32–0.83)		0.51 (0.30–0.87)	
Sex	Female	1.00	0.41	1.00	0.36
	Male	1.27 (0.72–2.23)		1.31 (0.73–2.37)	
Number of previous CD4 counts	0	1.00	0.04	1.00	0.01
	1	0.89 (0.57–1.40)		0.76 (0.48–1.22)	
	2+	0.49 (0.29–0.85)		0.43 (0.25–0.73)	
Social support – open with friends/family	Not at all	1.00	0.06	1.00	0.02
	A little	0.90 (0.52–1.57)		0.81 (0.45–1.43)	
	A lot	1.54 (0.93–2.55)		1.57 (0.94–2.62)	
Most recent partner HIV status	Positive	1.00	0.10	1.00	0.01
	Not known	0.78 (0.51–1.19)		0.77 (0.50–1.19)	
	Negative	1.78 (0.81–3.90)		2.40 (1.18–4.88)	
Number of children	None	1.25 (0.46–3.38)	0.03	0.68 (0.24–1.87)	0.04
	1 child	2.34 (1.32–4.14)		2.05 (1.14–3.69)	
	2 children	1.99 (1.13–3.50)		1.71 (0.94–3.09)	
	3 children	1.34 (0.69–2.60)		1.14 (0.57–2.30)	
	4+ children	1.00		1.00	

To explore the relationship between (a) openness with family and friends and LTFU (b) most recent partner HIV status and LTFU, post-hoc Competing Risks regression analyses examined interactions between openness with family and friends and most recent partner HIV status and the following potentially relevant psychosocial factors: (a) stigma (b) number of categories of people disclosed to. There were no significant interactions.

Post-hoc analyses also explored interactions between HIV partner disclosure (based on self-report of having disclosed to anyone, followed by identifying that a spouse/non-marital partner had been disclosed to) and: (a) openness with family and friends (b) most recent partner HIV status. There was an interaction between openness with family and friends and HIV partner disclosure that was close to significance (*p* = 0.06). To explore this further, a composite variable of openness with family and friends/partner HIV disclosure was created and included in multivariable analysis along with age, sex, number of previous CD4 counts, most recent partner HIV status and number of children (variables from the final model reported in Table 3). Higher LTFU rates were independently associated with younger age, fewer previous CD4 counts, most recent partner status being HIV-negative, and number of children (all with very similar estimates to those from the final model in Table 3), and the composite openness with family and friends/partner disclosure variable (Table 4). All categories of openness and partner disclosure appeared to be related to higher LTFU rates than being not at all open with family and friends and reporting no partner disclosure. The model with the composite openness/partner disclosure variable (Table 4) had a lower AIC value than the model presented in Table 3. Sub-analyses exploring the interaction between openness with family and friends and HIV partner disclosure using only those in a current partnership at recruitment (*n* = 185), showed that this interaction reached statistical significance (*p* = 0.04). There was not an interaction between most recent partner HIV status and HIV partner disclosure.

**Table 4:** Multivariable model of associations with loss-to-follow up in pre-ART care (*n* = 247) including composite social support and partner disclosure variable^a^ using Competing Risks regression

		Subhazard Ratio (95 % CI)	*P* value of association with LTFU (LRT)	Adjusted Hazard Ratio (95 % CI)	*P* value of association with LTFU (LRT)
Age	≤37	1.00	0.01	1.00	0.02
	>37	0.51 (0.32–0.83)		0.49 (0.28–0.87)	
Sex	Female	1.00	0.41	1.00	0.29
	Male	1.27 (0.72–2.23)		1.38 (0.76–2.52)	
Social support – open with friends/family and partner disclosure	Not at all open and not disclosed to partner	1.00	0.06	1.00	0.01
	Not at all open and disclosed to partner	3.40 (1.18–9.83)		3.03 (0.95–9.73)	
	A little open and not disclosed to partner	2.55 (0.82–7.96)		2.72 (0.86–8.64)	
	A little open and disclosed to partner	1.88 (0.65–5.49)		1.41 (0.44–4.53)	
	A lot open and not disclosed to partner	3.84 (1.30–11.31)		4.22 (1..47–12.11)	
	A lot open and disclosed to partner	3.48 (1.24–9.78)		3.12 (1.03–9.45)	
Number of previous CD4 counts	0	1.00	0.04	1.00	0.01
	1	0.89 (0.57–1.40)		0.71 (0.45–1.14)	
	2+	0.49 (0.29–0.85)		0.43 (0.25–0.73)	
Most recent partner HIV status	Positive	1.00	0.10	1.00	0.02
	Not known	0.78 (0.51–1.19)		0.74 (0.45–1.22)	
	Negative	1.78 (0.81–3.90)		2.18 (1.05–4.51)	
Number of children	None	1.25 (0.46–3.38)	0.03	0.69 (0.25–1.94)	0.02
	1 child	2.34 (1.32–4.14)		2.23 (1.21–4.10)	
	2 children	1.99 (1.13–3.50)		1.88 (0.99–3.54)	
	3 children	1.34 (0.69–2.60)		1.15 (0.55–2.39)	
	4+ children	1.00		1.00	

^a^Representing an interaction between openness with family/friends and partner disclosure

## Discussion

We assessed the relationship between psychosocial, demographic and clinical variables and LTFU in HIV-positive individuals in pre-ART care. As seen in previous studies, the LTFU rate (38.1 %) was higher than in those eligible for or initiating ART [4, 11]. Direct comparisons between pre-ART cohorts and ART cohorts are problematic, however, as loss to follow-up from ART is conditional on having started ART.

The association between younger age and higher LTFU rates is consistent with other studies on pre-ART LTFU [8, 33] and with studies on those ART-eligible [11, 34]. *How* age impacts upon LTFU needs clarification. For example, beliefs about the consequences of engaging in care might differ with age [35] or there may be greater competing demands that impact upon clinic attendance in younger than in older individuals. Competing demands life activities have been reported as a barrier to patient retention in other studies [36]. The relationship between younger age and higher LTFU rates (across studies that analyse age and calculate LTFU differently) may have clinical implications. Integrating pre-ART care with services specific to younger peoples’ needs (e.g., reproductive health services [37]), could be considered. Strategies that have a potentially beneficial effect on retention in the pre-ART phase, regardless of age (e.g., mobile phone prompts [38], cotrimoxazole prophylaxis [39], transport vouchers [40], home counselling [41], and case management [42]), could also be implemented.

The association between greater openness to family and friends and LTFU was also seen in a parallel cohort of ART-eligible individuals [11]. Being open with family and friends may mean that emotional support is offered by one’s social network rather than sought from clinic staff. Being open with family and friends may provide the opportunity for others to provide reassurance to the individual that clinic attendance is not yet necessary. Future research could assess social support more comprehensively, or could use a qualitative approach to explore these possibilities in more depth.

The close to significant interaction between openness to family and friends and HIV partner disclosure is worth further examination. Lower LTFU rates among those not open with family and friends and not having disclosed their HIV status to a partner, suggests that clinic attendance may provide an important source of social support for this group. There have been reported sex differences in patterns of HIV disclosure, with women more likely to disclose to multiple categories of people [43]. The relationship between LTFU and the combined openness and partner disclosure variable in our sample, however, remained once sex was controlled for (in a predominantly female sample).

Taken together, both the main effect of openness with family and friends and the interaction between openness with family and friends and partner disclosure suggest that social support needs may be salient even when medical needs are less pressing. Further investigation of the relationship between social support and LTFU may highlight the need to strengthen psychosocial support services in pre-ART care. In sum, clinic attendance may be motivated by both social and health needs.

Greater LTFU was associated with having an HIV-negative partner rather than a partner who is HIV-positive or of unknown status. It may be that HIV positive partners are better able to support ongoing clinic attendance or that having an HIV negative partner is a barrier to retention. Future qualitative studies could investigate this finding further. LTFU was independently associated with number of children. There was no clear linear pattern between the number of children and LTFU, however. The category with the lowest LTFU rate in the final model was having no children. The precision of this estimate was low, due to the small number of childless individuals. A greater sample size would allow for more exploration of this and other effects. LFTU was also associated with a history of previous CD4 tests. Individuals who were recruited when they had received their first CD4 count had greater LTFU than those who were already in HIV care and had received previous CD4 counts. This result was unsurprising as the latter individuals had already shown their willingness and ability to return for at least one repeated CD4 test in pre-ART care.

A number of variables (male sex, the receipt of government grants, never having been married and outward migration) were not significantly associated with LTFU in our analyses, despite being previously identified as associated in other studies in the literature, perhaps due to insufficient statistical power. Some of these variables may have been related to other exposures that were in the final model (e.g., outward migration and the number of previous CD4 counts). Sex has been associated with time to ART-eligibility [44] in the same cohort, and travel costs have been identified as the largest expense for those in pre-ART care in the same programme [45]. Being unmarried and outward migration have both been shown to be related to LTFU in other pre-ART samples [8, 10].

The LTFU rate is this study appeared to be lower than those reported for individuals with a CD4 count of >500 mm/cells^3^ in an earlier study on the programme population [8]. Directly comparing between the studies is difficult, however. We only recruited from three of the 17 clinics that offered HIV care in the local programme, specifically those that were most accessible. In addition, our study only included individuals who had returned for the CD4 count that determined their eligibility for ART. In the earlier study, all individuals were included regardless of whether they had returned for their CD4 test result. Although the definition of LTFU was comparable in both studies, our longer follow-up period will have made it more likely that some individuals will have experienced symptoms as their CD4 count dropped [46]. This may have prompted clinic attendance. Finally, our study was based on data from a later time period, during which guidelines for ART eligibility changed significantly. Alternatively, our lower LTFU rate may in part have been due to prompting associated with on-going participation in the prospective cohort study [12] that involved interviews every six months either in clinic or by phone. A more ideal study design would have been to prospectively follow individuals in pre-ART care through the programme only, however using routine programme data would have meant that other data available to explore as factors associated with LTFU would have been limited. Study retention was, however, separate from ART programme retention, and did not rely on clinic attendance. For example, 39 % of the 6 month cohort study interviews were not conducted at the clinic [12]. Our sample was similar in demographic characteristics to pre-ART patients in the HIV treatment and care programme as a whole, including the sex ratio disparity [8], and mortality rates were consistent with other pre-ART studies [4]. Post-hoc analysis showed no differences in estimates when we conducted the analysis separately for those participants without a previous CD4 count (*n* = 95) and those with a previous CD4 count (*n* = 152).

This study assessed a wider range of variables (particularly psychosocial variables) than other studies and was strengthened by a prospective cohort design with data on clinic attendance collected over a four-year period. Considerable effort was made to minimise ascertainment bias (e.g., participant tracking in the context of the prospective cohort study [12] and cross-checking outcomes between databases). The Competing Risks regression analysis gave similar results to those produced by using Cox regression. It is acknowledged, however, that there are limitations with using Competing Risks regression, which assumes conditional independence of different events.

Some of the variables measured at recruitment may have changed over the study duration (e.g., openness with family and friends). Both initial and intervening unmeasured factors may have influenced whether patients were LTFU at the end of the study period (e.g., beliefs about the consequences of attending clinic, symptoms, nature of the patient-provider relationship, mood, migration or alcohol use). Future studies could use a larger sample and a theoretical framework of engagement in care to select potentially explanatory variables, (e.g., specific beliefs about care [35]), that might be amenable to intervention.

## Conclusion

In summary, this study showed that being more open with family and friends, younger age, having fewer previous CD4 counts, number of children and most recent partner’s HIV negative status were independently related to higher rates of HIV treatment and care programme LTFU in individuals in pre-ART care. These relationships are important given the need to retain large numbers of HIV-positive individuals who are not ART-eligible (specifically those with a CD4 count of >500 cells/mm^3^) in long-term care. We also showed that even though the rates of LTFU may differ between those not yet eligible for ART and those who are eligible and/or on treatment, some of the predictors of LTFU may be similar [11].

### Ethical approval and consent to participate

Ethical approval was obtained from KwaZulu-Natal Department of Health, after review by the Biomedical Research Ethics Committee at University of KwaZulu-Natal (ref: BF083/08), and from London School of Hygiene and Tropical Medicine (ref 08/365). Written consent was given for participation with separate consent to link study data with HIV treatment and care programme and surveillance data.

### Consent for publication

Not applicable.

### Availability of data and materials

Data from this study are available from the Africa Centre data repository http://www.africacentre.ac.za/index.php/data-rep.
